# Too many, too soon? Challenges in medical school expansion in the United Kingdom

**DOI:** 10.1186/s12960-025-01038-8

**Published:** 2025-12-30

**Authors:** Tomas Ferreira, Alexander M. Collins

**Affiliations:** 1 Bristol Medical School, University of Bristol, 5 Tyndall Avenue, Bristol, BS8 1UD UK; 2 St Thomas’ Hospital, Guy’s and St Thomas’ Trust, London, UK; 3 Whipps Cross University Hospital, Barts Health NHS Trust, London, UK

**Keywords:** Health policy, Workforce planning, Medical education, Medical careers

## Abstract

The persistent shortfall in medical staffing in the UK has drawn renewed focus since the government first published its NHS Long Term Workforce Plan, in which it pledged to double the capacity of UK medical schools in the context of significant bottlenecks in doctors’ pathways to career progression. In this article, we challenge the viability of medical school expansion as a tool to combat the persistent workforce crisis in the NHS. Our critique contributes to the ongoing workforce planning debate, and highlights issues with clinical capacity, sustainability, and the preservation of educational standards. With an updated Long Term Workforce Plan forthcoming, we urge policymakers to implement an immediate moratorium on medical school expansion in the UK until these factors — and a viable future for the medical profession — can be guaranteed.

## Main text

### Introduction

The United Kingdom consistently ranks among the lowest of European OECD countries in terms of doctor-per-capita ratios, a disparity that has drawn considerable attention from policymakers and the media [1]. This shortfall, exemplified by more than 10,000 secondary care medical vacancies in England alone [2], has long been cited as a contributory factor to the oft-cited National Health Service (NHS) workforce crisis, a situation exacerbated by increasing demand for healthcare services, growing patient complexity, and the effects of chronic underfunding.

In response, the UK Government has outlined ambitious goals in the NHS Long Term Workforce Plan, seeking to double the number of medical school places to 15,000 per year by 2031 [3, 4]. The publication of the new 10 Year Health Plan for England outlines a revised strategy for medical school expansion, albeit with no specific mention of intake targets, to focus on bolstering schools, where widening participation efforts have previously been successful [5]. Though this may indicate more modest expansion of existing schools than originally proposed, there remain additional efforts to establish new medical schools in regions with historically poor doctor retention.

Though presenting an ostensibly straightforward solution to a complex problem, these proposals fail to address several systemic challenges underpinning the workforce crisis. The mismatch between workforce enlargement and existing infrastructure, persistent training bottlenecks, and the well-documented retention crisis among NHS doctors [6–8] raises concerns about the feasibility and long-term impact of such expansion. Moreover, the establishment of new medical schools—many with limited track records and resources—may intensify these issues. While accreditation is legally required, current evidence suggests the system is under significant strain. The GMC’s quality assurance framework for new schools involves self-assessment questionnaires, screening visits, annual follow-ups and final sign-off by Council, but each juncture is vulnerable to resource limitations (self-assessment questionnaires, visits, collating feedback) [9]. Meanwhile, the educator base itself is dwindling, as MSC data show 35% of clinical academics are aged over 55, reducing capacity for teaching and oversight [10]. Assessment practices already vary markedly between existing schools in volume, intensity and standard setting, and studies using common-content items reveal significant inter-school performance differences [11]. Taken together, these tensions raise the prospect that accreditation could be reduced from a rigorous safeguard to a procedural box-ticking exercise if expansion continues apace without commensurate investment. This risk is already evident, with some new medical schools enrolling students before securing full approval. Once cohorts exist, the GMC is under pressure to accredit to avoid disadvantaging them and negatively impacting their students, reversing the intended order of quality assurance and weakening regulatory leverage. Such practices risk eroding trust in UK medical qualifications and undermining the safeguards that protect patients and educational standards.

**Fig. 1:**
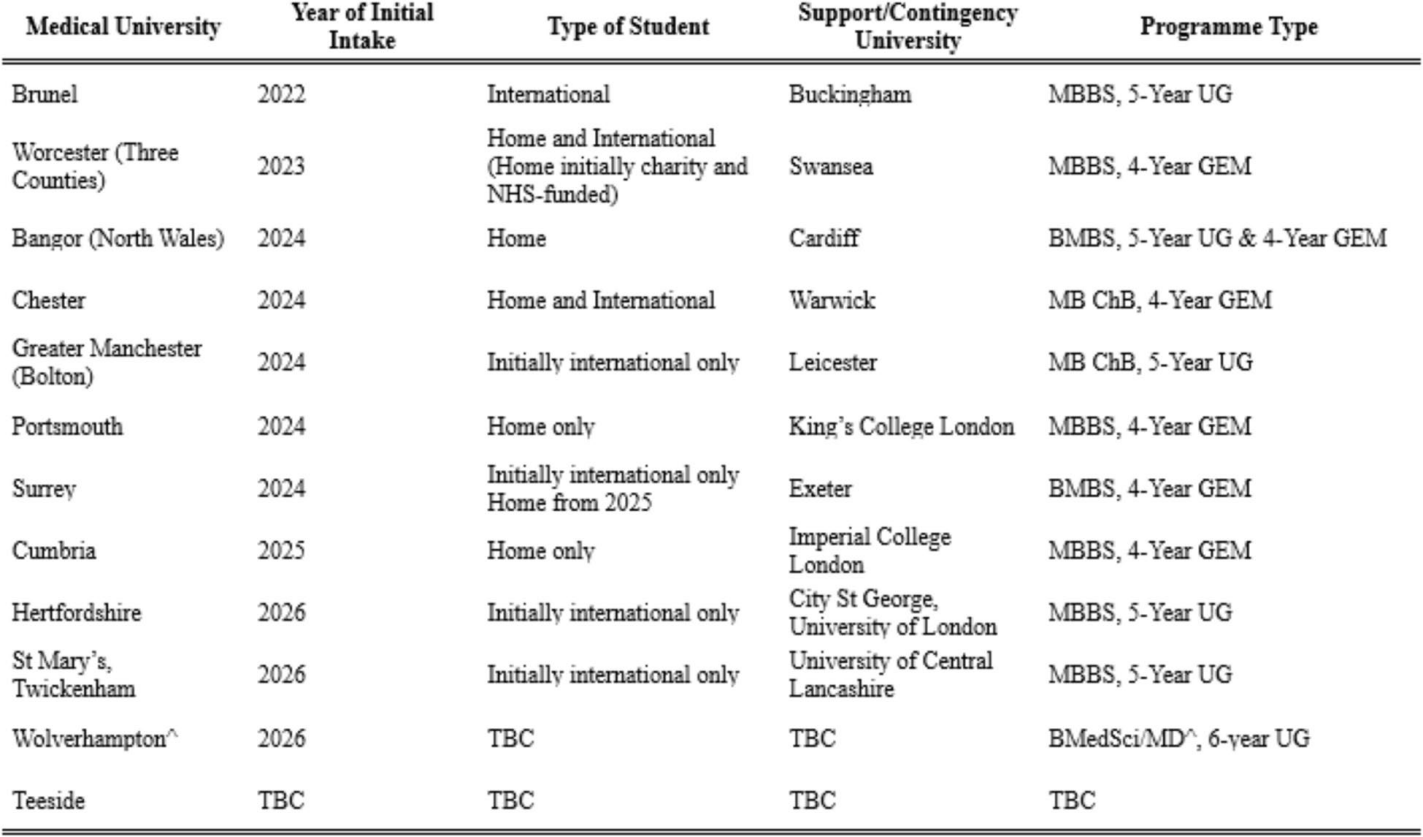
Overview of New and Planned Medical Schools in the UK. ^Proposal states would be run jointly by Wolverhampton University, UK, and American University of the Caribbean School of Medicine, Sint Maarten. *MBBS, MB ChB* Bachelor of Medicine, Bachelor of Surgery, *UG* Undergraduate, *GEM* Graduate-Entry Medicine, *BMedSci* Bachelor of Medical Sciences, *MD* Doctor of Medicine, *TBC* To be confirmed

This paper examines the implications of these workforce expansion plans, highlighting critical gaps in capacity, retention, and educational quality. Figure 1 provides an overview of the new medical schools established or proposed. These developments are critically analysed in the context of their potential to address, or aggravate, the ongoing NHS workforce challenges.

#### Do we have a shortage of doctors?

The prevailing narrative of a doctor shortage in the UK often overlooks the relationship between workforce size and healthcare infrastructure. While the UK trails behind many of its OECD counterparts by doctors per capita [1], it also lacks the hospital beds, operating theatres, and clinical capacity necessary to fully utilise an expanded medical workforce [12, 13]. This infrastructural deficit undermines the effectiveness of increasing medical student intakes to solve the NHS workforce crisis.

**Fig. 2:**
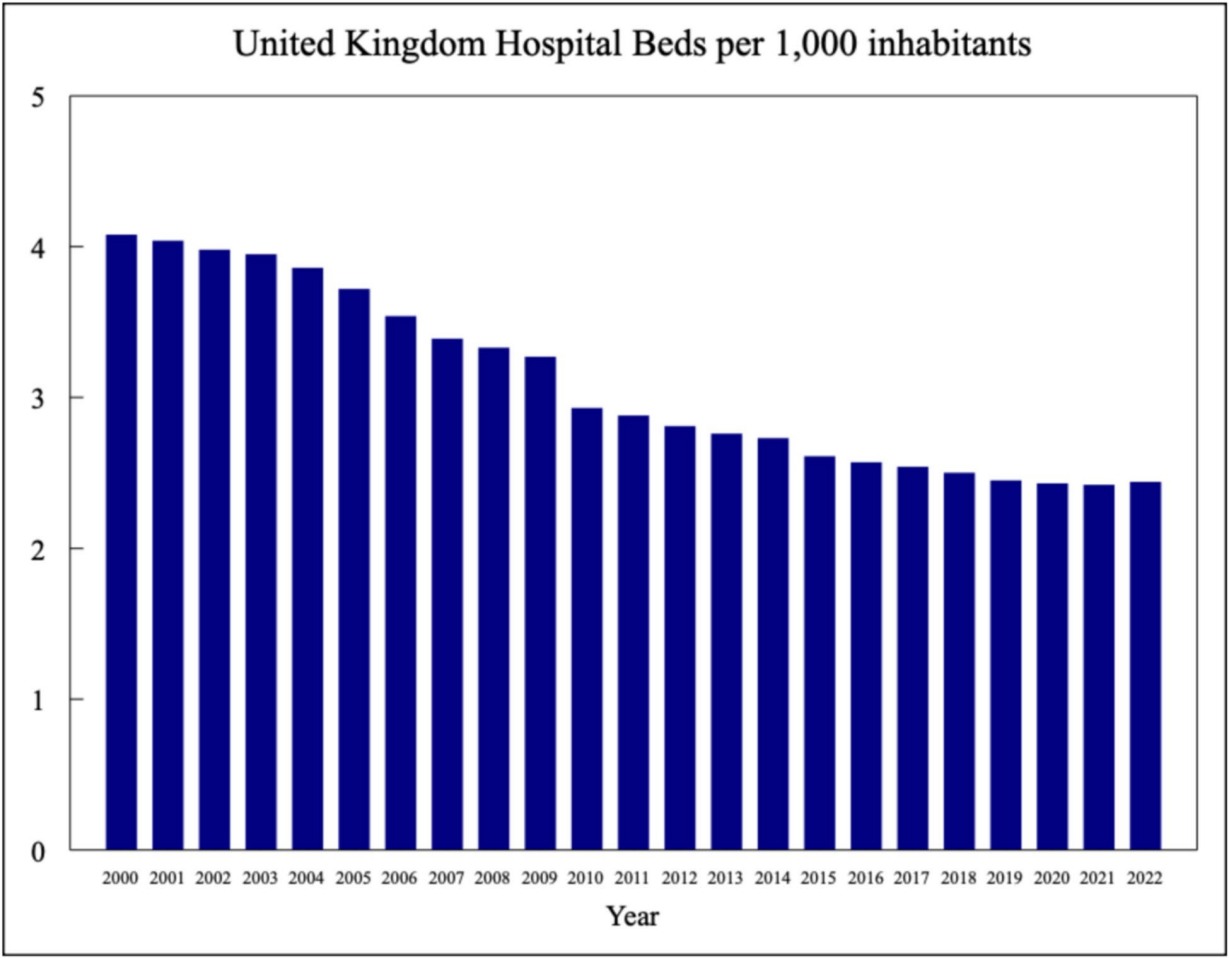
Decline in UK hospital beds per 1,000 population

The number of hospital beds in the UK has steadily declined over recent decades, reflecting broader trends in healthcare delivery but also highlighting capacity limitations. Although an imperfect indicator of healthcare delivery, given the role of outpatient care, among other factors, bed numbers provide a useful proxy when comparing the NHS to other systems. In 2000, the UK had 3.1 hospital beds per 1000 people; by 2021, this figure had dropped to 2.4 beds per 1000 people: a stark contrast to European averages, where countries such as Germany maintain over 8 beds per 1000 people [12]. Figure 2 illustrates these trends, demonstrating the UK’s decline in hospital bed availability. Increasing the number of doctors without corresponding investment in infrastructure risks overwhelming already strained resources and reducing the quality of patient care.

Another overlooked dimension is the severe bottleneck in postgraduate training. The limited availability of foundation and specialty training positions already presents a significant barrier to career progression. Competition for entry into specialty training has risen dramatically, with many disciplines seeing record levels of applicants per post [14]. This mismatch has left increasing numbers of qualified doctors without clear training pathways, heightening dissatisfaction and attrition within the medical workforce [15]. This bottleneck is already apparent among undergraduates: in a recent national survey, only 23.1% of UK medical students felt confident in securing a specialty training post after completing the foundation programme [16]. 

#### Capacity for clinical training

The UK’s medical academic workforce is ageing, with a substantial proportion of educators nearing retirement and insufficient recruitment of new academic staff to replace them [10]. Compounding this issue is the static number of educators, which has remained largely unchanged despite rising student numbers [17]. This mismatch already exerts considerable strain on the current workforce, with high workloads contributing to burnout and attrition among educators. Without parallel investment in recruitment and educator retention, the additional strain may drive more teaching staff out of academia, creating a feedback loop of worsening capacity and declining educational standards.

The establishment of new medical schools raises further concerns about the potential dilution of educational quality. Likely subject to the broader crisis in higher education funding in the UK [18], new institutions may lack the infrastructure, resources, and experienced faculty to deliver rigorous medical training. Working hastily to meet expansion targets, there is a risk that poorly resourced schools will prioritise increasing student numbers over maintaining high standards of education. This could undermine the long-term viability of medical schools which lack the reputation and resources to recruit from the depleted pool of medical educators.

Key risks to educational standards include recruitment of underqualified educators due to shortages, inadequate facilities and resources for clinical placements, and substandard accreditation processes. Shortages of experienced clinical educators may force new schools to rely on less qualified or less experienced staff, affecting the quality of teaching and mentorship available to students. Many new medical schools may face challenges in securing access to clinical placements and essential training facilities, limiting students’ exposure to real-world clinical environments. The urgency to establish new institutions may also result in accreditation processes becoming less stringent, allowing schools to operate without meeting established benchmarks for quality.

These educational issues collectively risk replacing quality with quantity, producing graduates ill-equipped for the demands of clinical practice. Indeed, inadequate training environments and mentorship could have far-reaching consequences, including poorer patient outcomes and reputational damage to UK medical qualifications.

#### A retention crisis

High levels of stress, burnout, and dissatisfaction among NHS doctors contribute to significant rates of attrition, a problem that is compounded by the highly competitive nature of career progression. As competition ratios for specialty training positions reach record levels [19], the rising numbers of unplaced graduates highlight a system unable to accommodate the growing workforce [20]. Unemployment and career stagnation compound this dissatisfaction.

In 2023, over 11,000 doctors relinquished their licence to practise in the UK [15]. This, amidst over one-third of students stating an intention to leave the NHS within 2 years of graduation, whether through emigration or exiting the profession entirely [6], highlights the urgent need to prioritise strategies emphasising retention over expansion. Increasing graduate numbers alone will not resolve the systemic factors driving professionals away—without intervention, the NHS risks further entrenching its "leaky bucket" phenomenon, where investments in training new doctors fail to yield a sustainable workforce.

Retention challenges also undermine broader aims of workforce expansion. Many graduates, unable to secure fulfilling roles within the NHS, will seek opportunities abroad, effectively staffing other healthcare systems. This represents a waste of taxpayer resources, and further compounds morale issues among those who remain, with the burden of unmet staffing rising.

### The role of new medical schools

The NHS Long Term Workforce Plan, in addition to expanding the delivery of traditional medical curricula, posed two new modes of medical education delivery. The first pathway proposed, which was piloted and later suspended indefinitely, was the medical doctor degree apprenticeship [3, 4, 21].Though offering a non-traditional pathway into medicine, there were significant concerns about apprenticeships’ potential impact on educational standards and equity within the profession, with the British Medical Association formally voting to oppose their rollout [22]. Similarly, controversial, though not yet abandoned, was the proposal to introduce accelerated, 4-year undergraduate medicine courses [23, 24].

The apprenticeship model presented significant risks to educational standards, equity, and the quality of training, raising fears of a two-tier system, where apprentices’ qualifications may have lacked international recognition, disproportionately disadvantaging students from lower socioeconomic backgrounds [25]. It is encouraging that the apprenticeship scheme will no longer proceed; however, the episode underscores the importance of rigorous oversight to ensure new institutions uphold high educational standards.

Unlike the apprenticeship model, the proposal for 4-year undergraduate medicine has not been formally halted, though it was notably absent from the recent 10 Year Health Plan for England and there is no published safety case or piloted evaluation demonstrating equivalence on core outcomes. While framed as a means of accelerating workforce supply, such a shift risks creating a two-tier system within the profession, separating graduates of traditional 5- or 6-year programmes from those entering via compressed or alternative routes. A shortened course risks weaker international portability while reducing clinical exposure, narrowing valuable assistantships and electives, and straining already limited supervisor capacity. In addition, the financial and logistical pressures of delivering a condensed curriculum will likely incentivise universities to cut non-clinical teaching time—either eroding the depth of pre-clinical scientific foundations and further entrenching a protocol-driven style of medicine over true clinical reasoning, or sacrificing student-selected components, research projects, and longitudinal electives that cultivate intellectual curiosity and academic leadership. These trade-offs raise serious concerns about the calibre and versatility of future graduates and heighten patient safety and assessment quality risks at the point of provisional registration. The model is also likely to disadvantage widening participation students and does nothing to relieve the fixed bottlenecks in foundation and specialty training, so graduates would simply reach a longer queue sooner. On balance, the policy diverts resources from the real rate-limiters of workforce supply, namely, clinical infrastructure, funded posts, and retention.

Moreover, many new medical schools have a disproportionately high intake of international medical students compared to established institutions, with most inaccessible—at least initially—to students of home fee status (Fig. 1) [26]. While this provides a significant revenue stream through higher tuition fees, it raises critical concerns regarding their long-term contribution to the UK doctor workforce. International students may be less likely to remain in the UK long-term, meaning these schools’ added value—and use of limited clinical placement capacity—may be further questioned, especially as medicine continues to attract a large surplus of home applicants annually [6]. Some institutions may, therefore, adopt this model initially to gain credibility and secure funding for home student places, but this reliance on international students prioritises institutional income over solving the NHS's pressing workforce challenges, further undermining the purported goals of workforce expansion.

#### Learning from international case studies

International examples provide critical lessons for the UK as it seeks to expand its medical education capacity. These case studies underscore the risks associated with rapid, poorly planned medical school expansion and highlight the importance of addressing systemic issues alongside numerical enlargement.

In India, a rapid proliferation of private medical colleges led to significant variability in the quality of education. Many of these institutions suffer from insufficient regulation and a lack of oversight, leading to substandard training for graduates [27]. As a result, large numbers of medical graduates face unemployment or underemployment, unable to secure suitable positions within the healthcare system. To address this issue, the Indian Medical Association has explored initiatives to deploy graduates internationally, effectively exporting the burden of excess graduates [28]. This approach exemplifies the consequences of expanding medical education without ensuring the alignment of training quality with workforce needs and capacity.

South Korea provides another cautionary example. In response to perceived doctor shortages, the government enacted a policy to increase annual medical student intake by 60%. However, this move triggered widespread industrial action among resident doctors, with thousands resigning in protest [29]. The strikes highlighted several critical concerns, including risks to training standards, insufficient resources to accommodate the additional students, and a fundamental misalignment between workforce needs and the number of graduates being produced. Rather than addressing systemic issues such as retention and regional maldistribution of doctors, the policy inflamed existing tensions within the healthcare system, demonstrating the risks of pursuing numerical expansion without a comprehensive strategy for integration and sustainability.

### Recommendations

**Fig. 3:**
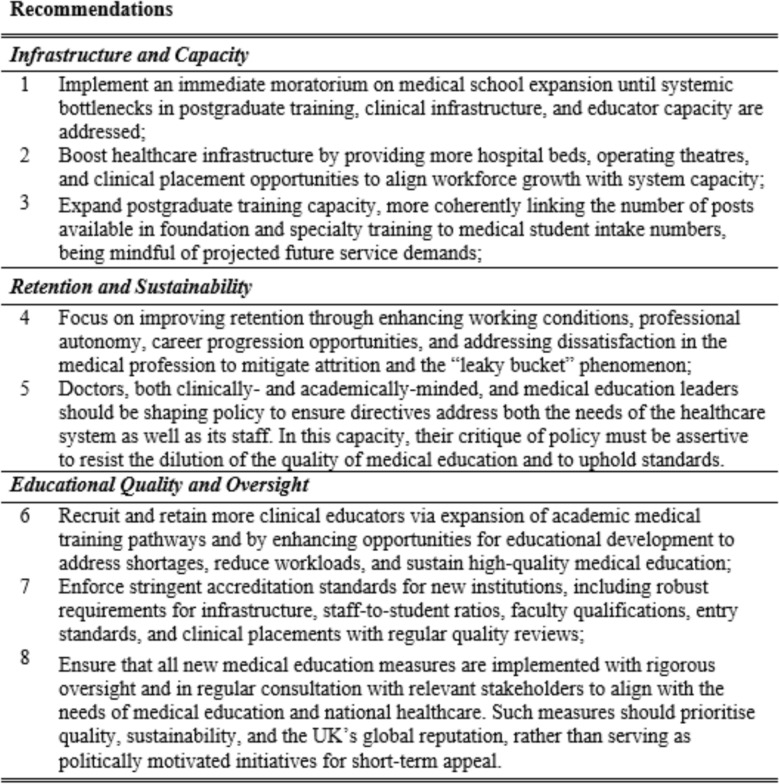
Recommendations for addressing challenges in medical school expansion and the NHS workforce crisis

Figure 3 depicts includes our recommendations. We hope their implementation would support the NHS to address the root causes of its workforce crisis while preserving the quality, sustainability, and global reputation of UK medical education.

### Conclusion

Expanding medical school places without addressing systemic bottlenecks risks intensifying the NHS workforce crisis. Insufficient infrastructure, limited training capacity, and poor retention measures undermine these plans’ effectiveness. The rapid proliferation of new medical schools, particularly from institutions without established reputations or adequate resources, raises legitimate concerns about maintaining the high standards of UK medical education. These developments threaten the global standing of UK-trained doctors if robust oversight and quality assurance are not prioritised. Lessons from international examples underscore the dangers of poorly planned growth. Sustainable workforce solutions require prioritising retention, investing in infrastructure, and ensuring rigorous educational standards to secure the future of UK healthcare.

## Data Availability

No data sets were generated or analysed during the current study.

## Abbreviations

OECD: Organisation for Economic Co-operation and Development
NHS: National Health Service
